# RESULTS OF AN ONLINE CAREGIVING DURING CRISIS COURSE

**DOI:** 10.1093/geroni/igad104.2568

**Published:** 2023-12-21

**Authors:** Carolyn Clevenger, Fayron Epps, Melinda Higgins, Glenna Brewster, Kirandeep Raina, Kenneth Hepburn

**Affiliations:** Nell Hodgson Woodruff School of Nursing, Emory University, Atlanta, Georgia, United States; Emory University, Atlanta, Georgia, United States; Emory University, Atlanta, Georgia, United States; Emory University, Atlanta, Georgia, United States; Atlanta, Atlanta, Georgia, United States; Emory University, Atlanta, Georgia, United States

## Abstract

Caregiving during Crisis (CDC) is an online, asynchronous, self-paced course for dementia family caregivers. The course was developed to enhance caregivers’ sense of mastery for their role as systems navigator with the secondary effects of improving their own and their person’s quality of life. CDC was drafted by subject matter experts and finalized by family caregivers. The final version focused on three main topics: Safe Home; Navigating the Healthcare System; and Managing the Day divided into 54 short interactive segments. One hundred caregivers were enrolled in a randomized waitlist control group design, with measures of mastery and quality of life at three timepoints over 16 weeks. We employed multi-level mixed effects models (MLM) with random (participant) and fixed (group) effects to test linear (or possibly non-linear) trajectories of change for the immediate and waitlist groups and model group X time and group*time interactions. Of 100 consented participants (50 in immediate and 50 in waitlisted group), 76 completed the midpoint study activities (38 in each group). For the immediate group, there was an increase in mastery such that by the midpoint at 2 months, the caregivers in the immediate group had significantly higher mastery scores (p=.039) than those in the waitlist group, with a moderate effect size (d=0.501) difference. Fourteen course participants completed semi-structured interviews. Their responses to the course were very positive, noting it was well organized, logical, and not too technical. They also indicated a need to include guidance for interactions with legal, regulatory, social service, and financial systems.

